# Association of decreased *triadin* expression level with apoptosis of dopaminergic cells in Parkinson’s disease mouse model

**DOI:** 10.1186/s12868-021-00668-7

**Published:** 2021-11-04

**Authors:** Min Hyung Seo, Sabina Lim, Sujung Yeo

**Affiliations:** 1 College of Korean Medicine, Sangji University, #83 Sangjidae-Gil, Wonju, Gangwon-Do 26339 Republic of Korea; 2 WHO Collaborating Center for Traditional Medicine, East-West Medical Research Institute, Kyung Hee University, Seoul, 02447 Republic of Korea; 3 Department of Meridian & Acupoint, College of Korean Medicine, Kyung Hee University, #47 Kyungheedae-gil, Dongdaemun-gu, Seoul, 02453 Republic of Korea; 4 Research Institute of Korean Medicine, Sangji Univeristy, 26339 Wonju, Republic of Korea

**Keywords:** TRDN, Parkinson’s disease, Apoptosis, MPTP, SH-SY5Y

## Abstract

**Background:**

Parkinson’s disease (PD) represent a loss of dopaminergic neurons in the substantia nigra (SN) of the midbrain. However, its cause remains unknown and Triadin (TRDN) function in the brain is also unknown. To examine the relationship between *TRDN* and PD, the expression levels of protein related to PD in *TRDN* knockdown status were studied in the SH-SY5Y cells. Cell viability and apoptosis were assessed to examine the apoptosis effect on dopaminergic cells by decreased TRDN, and the levels of the proteins related to apoptosis were also confirmed.

**Results:**

This study confirmed decreased TRDN expression level (P < 0.005) at the SN in a 1-methyl-4-phenyl-1,2,3,6-tetrahydropyridine-induced PD mouse model and identified the functional features of TRDN. Our results showed a relationship between TRDN expression and PD in that reduced TRDN level induced PD-like characteristics. Interestingly, there was TRDN expression in the regions where dopaminergic cells are in the SN, and the expression patterns of TRDN and tyrosine hydroxylase (TH) were similar. Decreased TRDN level also induced apoptotic characteristics and the Fluorescence-activated cell sorting analysis results showed that apoptosis increased (P < 0.05) as the TRDN small interfering RNA concentration increased. The cytotoxicity assay revealed that cell viability also decreased (P < 0.0005) in the same condition as that in the Fluorescence‐activated cell sorting analysis.

**Conclusions:**

Decreased TRDN level could be related with the apoptotic death of dopaminergic cells at the SN in PD, and TRDN administration could give a positive effect on PD by reducing apoptotic cell death.

**Supplementary Information:**

The online version contains supplementary material available at 10.1186/s12868-021-00668-7.

## Introduction

Parkinson’s disease (PD) is the second most common degenerative brain disease in the elderly people [1]. PD causes clinical symptoms such as tremor, bradykinesia (slow movement), muscle rigidity, and postural instability. Some patients have dementia, depression, and anxiety [2].

In the brain, the pathological features show a loss of dopaminergic neurons in the substantia nigra pars compacta (SNpc) of the midbrain [3]. Furthermore, the level of tyrosine hydroxylase (TH), which catalyzes tyrosine to form L-DOPA, is decreased in the striatum (ST) and the substantia nigra (SN) [4].

The cause of PD is not known yet, but the conjectured causes of PD are oxidative stress, mitochondrial dysfunction, endoplasmic reticulum (ER) stress, and genetic factors such as familial type [4, 5]. Approximately 95% of PD cases is sporadic PD. Genetic PD due to genetic mutation of α-synuclein, Parkin, and Dj-1 accounts for 5% of all PD cases [2]. Meanwhile, exposure to 1-methyl-4-phenyl-1,2,3,6-tetrahydropyridine (MPTP) causes selective dopaminergic neurodegeneration of PD not only in human but also in mice. Therefore, MPTP is most commonly used to make an animal model of PD for study [2].

*Triadin* (TRDN) was mostly studied in cardiac and skeletal muscles. Several isoforms of TRDN are identified, and the function of TRDN inhibits Ca^2+^ release from the depolarization-induced sarcoplasmic reticulum (SR). In addition, TRDN-deficient mice had a reduced Ca^2+^ storing size of the SR [6]. Therefore, we could deduce that TRDN expression is closely related to Ca^2+^ regulation. When the muscle contracts, Ca^2+^ release from the SR is generated by plasma membrane depolarization. Accordingly, *TRDN* deletion was also reported to cause impaired muscle function [7]. Furthermore, in the previous study, we observed decreased expression of TRDN in muscle in a mouse model of Parkinson’s disease [8].

However, the function of TRDN and the relationship between TRDN and PD in the brain have not been reported. We hypothesized that reduced expression of *TRDN* may be associated with pathological features of PD in the brain. Thus, in this research, we examined TRDN expression in relation to the pathological features of PD and investigated how TRDN downregulation affects neurons in SH-SY5Y cells using small interfering RNA (siRNA) transfection. Moreover, on the basis of reported literatures, apoptosis is regarded as degradation of neurons in PD [9, 10]. Therefore, the relationship between TRDN and PD was studied in terms of apoptosis.

## Materials and methods

### MPTP mouse model of Parkinson’s Disease

Six-week-old male inbred C57BL/6J mice (20–22 g; DBL, Korea) were injected with MPTP-HCL (20 mg/kg of free base; Sigma, USA) in 100 µL phosphate-buffered saline (PBS) intraperitoneally once a day for 4 weeks in MPTP group (n = 6) whereas mice were injected with 100 µL PBS in control group (n = 6). The day after the final injections, mice were anesthetized using Alfaxan (4 mL/kg; Careside, Korea) and perfused transcardially with cold PBS. In this study, all animal experiments were approved from Sang Ji University Animal Experimentation Committee.

### RNA extraction and microarray analysis

RNA extraction and microarray analysis proceeded in the same procedure described in the previous study [11, 12]. The differentially expressed genes (DEGs) that satisfied the conditions of the fold change cutoff (1.5) and the Student *t*-test significance criterion (p < 0.05) were identified using the DEG-finding module [13].

### Immunohistochemistry

The procedures to obtain brains of 4-week MPTP Parkinson’s disease mouse models for immunohistochemical analysis were described well in previous research report [8]. The procedures were followed to investigate TH in SN regions. Mouse anti-TH antibody (1:200; Santa Cruz Biotechnology, USA) were used for incubation overnight at 4 °C. On the other hand, the procedures were followed until blocking step in observation of TRDN in SN. When observing TRDN, sections encompassing the SN regions were incubated in blocking buffer (1% bovine serum albumin [BSA] and 10% goat serum in PBS) for 1 h. Rabbit anti-TRDN antibody (1:200; MybioSource) were used for incubation overnight at 4 °C. And then, the sections were treated with biotinylated anti-rabbit IgG and avidin–biotin–peroxidase complex and were observed through reaction with diaminobenzidine–hydrogen peroxide solution.

### Cell lines and cultures

Cells from the SH-SY5Y cell line were cultured in standard culture conditions (5% CO_2_, 37 °C). Minimum essential medium (MEM; Welgen, Namcheon-myeon, South Korea) containing 10% fetal bovine serum (BioWest) [11], 100-U/mL penicillin-streptomycin (Gibco; USA), and 0.1 mM nonessential amino acids (Gibco, USA).

### Small interfering (si)RNA knockdown

To downregulate the expression of TRDN and examine the effects, siRNA against TRDN (5′-UC AUG UGG GUA GAC UCA GU-3′) and negative control duplexes (siRNA, 5′-UUC UCC GAA CGU GUC ACG UTT-3′) were used [11], and the siRNAs were obtained from Bioneer Inc., Korea. SH-SY5Y cells incubated in Opti-MEM medium at least 1 day before siRNA transfection. When transfection starts, the density of SH-SY5Y cells was 70%. Transfection reagent (Promega, USA) was used in a 3.5:1 transfection reagent-to-duplex RNA ratio in Opti-MEM (Gibco) medium during 24 h transfection.

### Western blotting

The SH-SY5Y cells were homogenized in 20 mM radioimmunoprecipitation assay buffer (RIPA) on ice for 20 min after PBS washing briefly [8, 11]. When the SN tissues (n = 3) were homogenized in 20 mM RIPA using a sonicator (Qsonica Q55; USA) and incubated on ice for 20 min. After centrifugation at 12,000 rpm at 4 °C for 15 min, supernatant samples of equal protein concentration were separated using 4–15% sodium dodecyl sulfate-polyacrylamide gel electrophoresis (SDS-PAGE) and then transferred to polyvinylidene difluoride (PVDF) membranes (Pall Life Science; USA) [11]. The membranes were blocked with 3% BSA in 0.1% Tris-buffered saline (TBS; 20-mM Tris–HCl [pH 7.5] and 150 mM NaCl containing 0.1% Tween-20, TBST) at room temperature for 1 h and then incubated with primary antibody overnight and then washed with 0.1% TBST [11, 13]. The membrane was also incubated with secondary antibody for 1 h and washed with 0.1% TBST. Rabbit anti-TRDN (1:2000; MyBioSource), rabbit anti-TH (1:5000; Abcam), rabbit anti-β actin (1:5000; Cloud Clone), rabbit anti-Bax (1:1000; Abcam), rabbit anti-Bcl-2 (1:5000; Abcam), and mouse anti-β actin (1:5000; Santa Cruz Biotechnology) were used as primary antibodies. Goat anti-mouse IgG (HRP; suitable ratios; Abcam) and goat anti-rabbit IgG H&L (HRP; suitable ratios; Santa Cruz Biotechnology) were used as secondary antibodies. The antigen-antibody complexes were visualized using ECL detection reagents (GE Healthcare; USA) by Amersham Imager 680 (GE Healthcare).

### Cytotoxicity assay

The cytotoxicity assay was performed to know how decrease TRDN level affects cells. Cell viability and cytotoxicity were examined using EZ-CYTOX (DoGenBio, Korea). The recommended protocol was followed, and cell viability was measured after TRDN siRNA(siTRDN) treatment for 24 h. SH-SY5Y cells were treated with various concentration of siTRDN (5, 10, 25, 50, and 100 nM) and 100-nM negative control siRNA.

### Fluorescence-activated cell sorting analysis

Fluorescence-activated cell sorting (FACS) analysis was conducted to do analysis of apoptosis in cells, according to siTRDN treatment. siTRDN treatment was performed in a same way with Cytotoxicity assay. Control group was added to do compensation and nothing was treated in the control group cells. Cells were collected with cell scraper and washed with PBS twice in 2500 rpm, 4 °C for 5 min. Cells were resuspended with binding buffer in 10^6^ cells/mL and 100 µL (10^5^ cells) was aliquot. Fluorescein(FITC)-annexin V and propidium iodide(PI) was treated following suggested protocol of manufacturer (BD Parmingen^TM^). Samples were diluted 5 times with binding buffer and detection was performed with cytoFLEX (Beckman Coulter Life Sciences; US).

### Statistical analysis

For statistical analysis, Student’s t-test and analysis of variance (ANOVA) in SPSS 25 (Released 2017, PASW statistics for Windows, version 25.0, Chicago: SPSS Inc.) were used. P values less than 0.05 are regarded as significant values and P < 0.05 values are expressed in figures. All values expressed as mean ± SEM.

### Imaging software

ImageJ software was used for image processing.

## Results

### Observations of TRDN and TH in substantia nigra of MPTP-induced PD mouse model

**Fig. 1:**
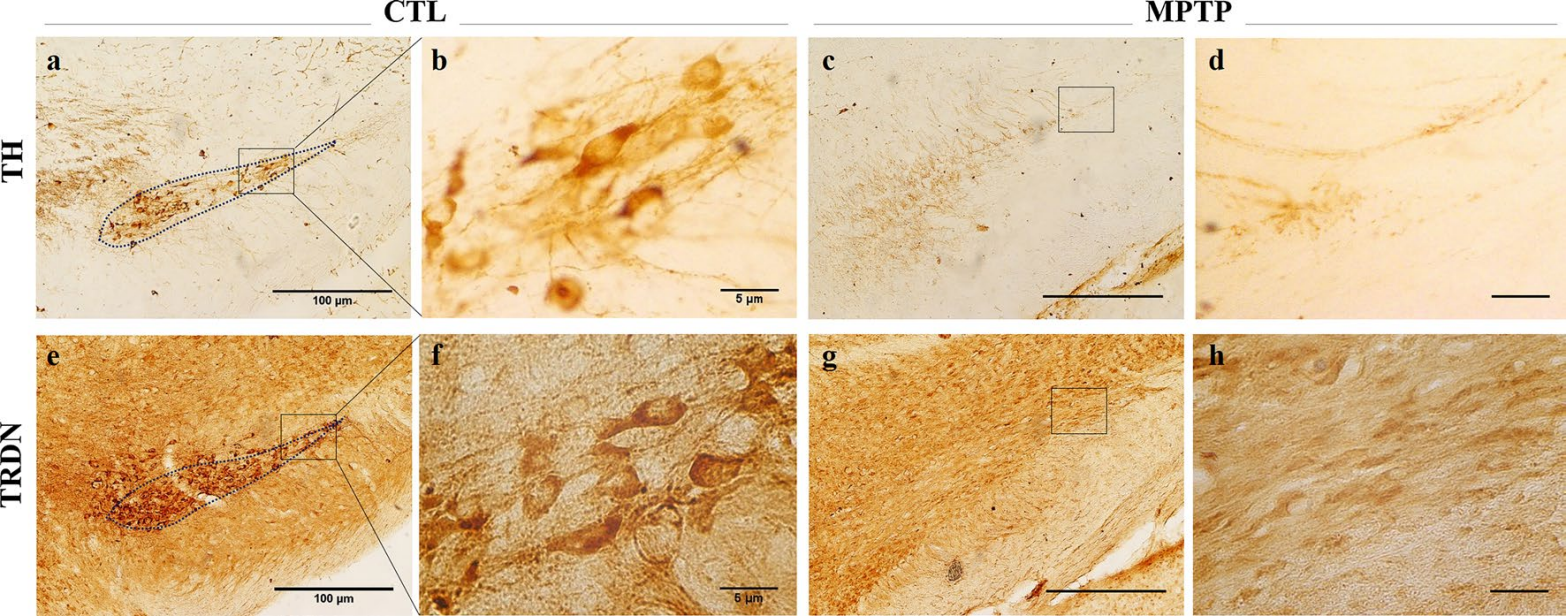
Changes and similarity of tyrosine hydroxylase (TH) and TRDN in dopaminergic neurons in substantia nigra (SN) related with Parkinson’s disease (PD). Immunohistochemistry results of TH and TRDN in SN regions of the control (CTL) group (**a**, **b**, **e**, **f**). The expression pattern of TH and TRDN in SN is similar (dotted lines in a and e; scale bar = 100 μm), and rectangular boxes in a and e panels are 10 times magnified (**b**, **f**; scale bar = 5 μm). TH and TRDN in SN regions of the MPTP group are also observed (**c**, **d**, **g**, **h**). Similarly, in the MPTP group, TH and TRDN expressions were much lesser than those in the SN region of the CTL group (**a**, **c**, **e**, **g**; ×100). Rectangular boxes in **c** and **g** panels are magnified (**d**, **h**; ×1000)

To make a MPTP-induced PD mouse model, mice were injected with MPTP-HCL (20 mg/kg) every 24 h for 4 weeks. Immunohistochemistry (IHC) using 3,3-diamino-benzidine was also performed to check the expression patterns in TRDN and TH levels in the SN region (Fig. 1). It was confirmed that TRDN which has not been known well to be expressed in the brain was expressed in the SN region of brain (Fig. 1e–h). Furthermore, the expression pattern of TRDN almost coincides with the re-gion of TH expressed in dopaminergic neurons of SN interestingly (Fig. 1a, e, b, f). In MPTP group, decreased TH level and dopaminergic cells in SN were also confirmed (Fig. 1a, c), and this means that the MPTP-induced PD mouse mod-el was made well. TRDN expression also decreased in MPTP group surprisingly (Fig. 1e, g).

### Changes of TRDN and TH in substantia nigra

**Fig. 2:**
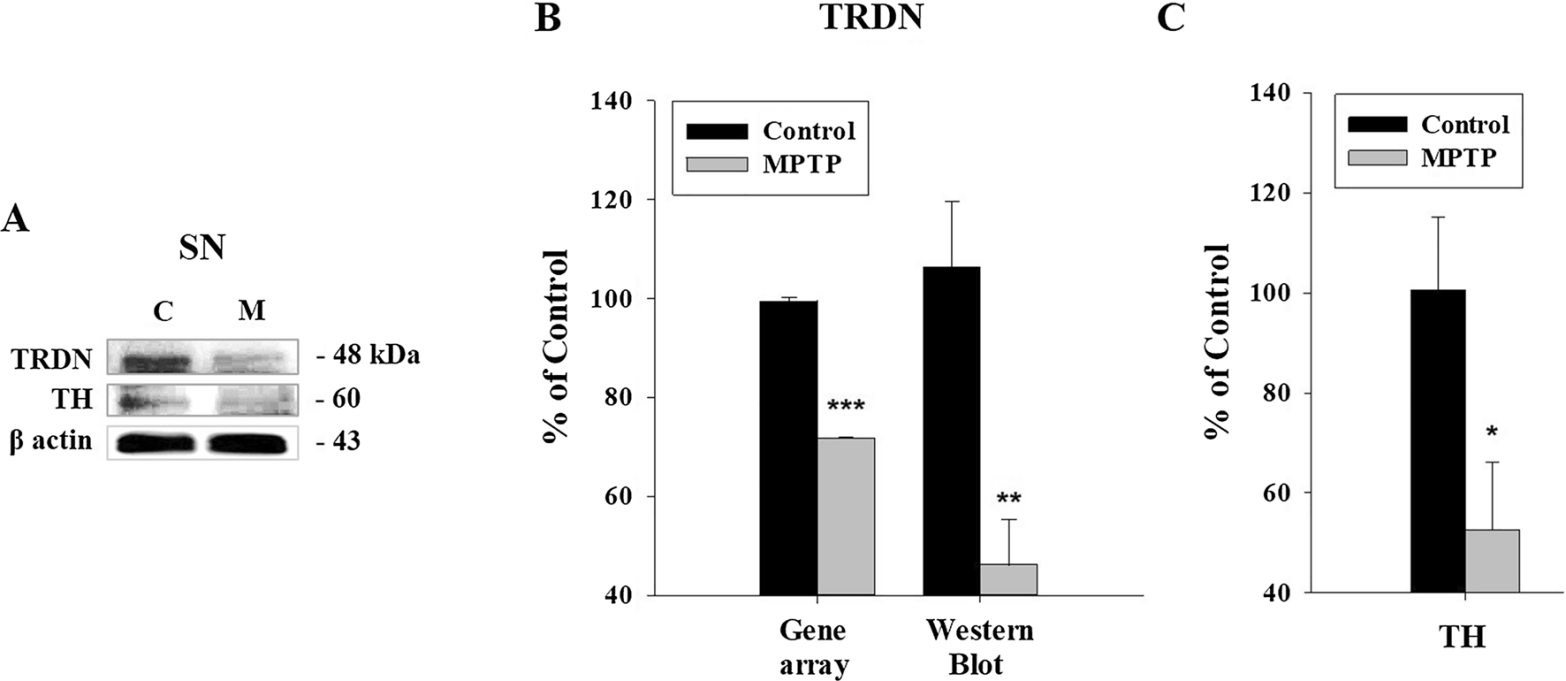
Changes of TRDN and tyrosine hydroxylase (TH) expression in substantia nigra (SN) of Parkinson’s disease (PD) induced mouse.** A** Western blot (WB) results of TRDN and TH at the SN region in the control (**C**) and MPTP (M) groups. The TRDN levels decreased in the MPTP group. **B** WB and Gene array results of TRDN in SN are shown in the graph. The TRDN gene expression level decreased. **C** WB results of TH in SN are shown in the graph. TH expression level decreased in SN of MPTP group (*P < 0.05, **P < 0.005, ***P < 0.0005)

The results of the gene array using the SN portion of the mouse brain confirmed that the *TRDN* gene expression level decreased in Fig. 2B. Furthermore, WB was performed to check the expression levels of TRDN and TH by extracting brain tissue, especially SN, from each group. The results are shown in Fig. 2A. As with the gene array results, the WB results show decreased expression levels of TRDN (Fig. 2A, B). These results confirmed the decrease in TRDN level in the MPTP-induced PD mouse model one more time. As results are shown in Fig. 2A, C, the WB results show decreased expression levels of TH in the MPTP group (Additional file 1).

### Observations of TRDN and TH in substantia nigra using immunofluorescence

**Fig. 3:**
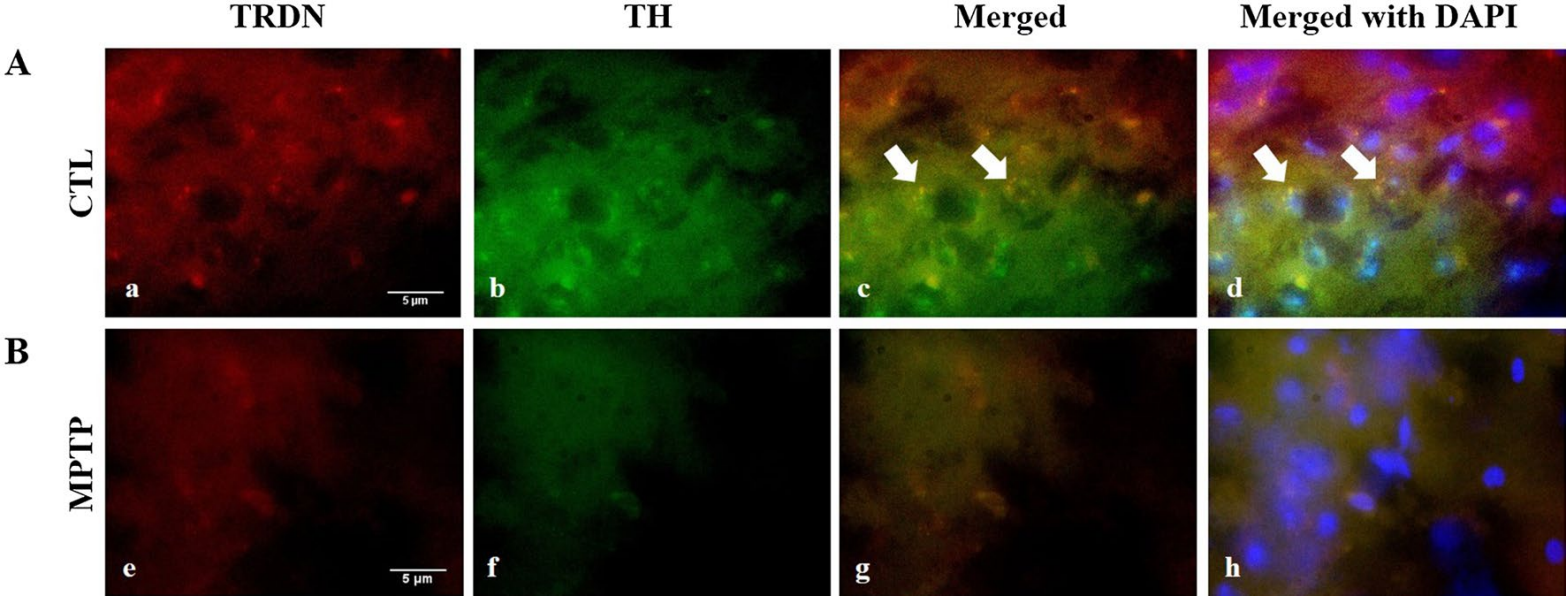
Double immunofluorescence labeling in substantia nigra (SN).** A **Control (CTL) group.** B **MPTP group. Double immunofluorescence was performed with antibodies against TH and TRDN in control (CTL; panels **a**–**d**) and MPTP (panels **e**–**h**). TH and TRDN were merged (**c**, **g**) and the levels of TH and TRDN decreased significantly in MPTP group. DAPI staining was also merged in control (panel **d**) and MPTP (panel **h**) groups. Scale bar = 5 μm

To observe the TRDN and TH in the SN of the control and MPTP induced-PD model groups simultaneously, immunofluorescence was performed. TH was stained with FITC, and TRDN was stained with TRITC. The results showed that TH and TRDN were merged successfully in the control group (Fig. 3c). On the other hand, in the MPTP group, the levels of TH and TRDN decreased significantly (Fig. 3e, f). Therefore, TRDN and TH interact with each other under the normal condition, but when PD develops, the TRDN level decreases and so does TH.

### Changes in factors related to apoptosis according to the alteration of TRDN

**Fig. 4:**
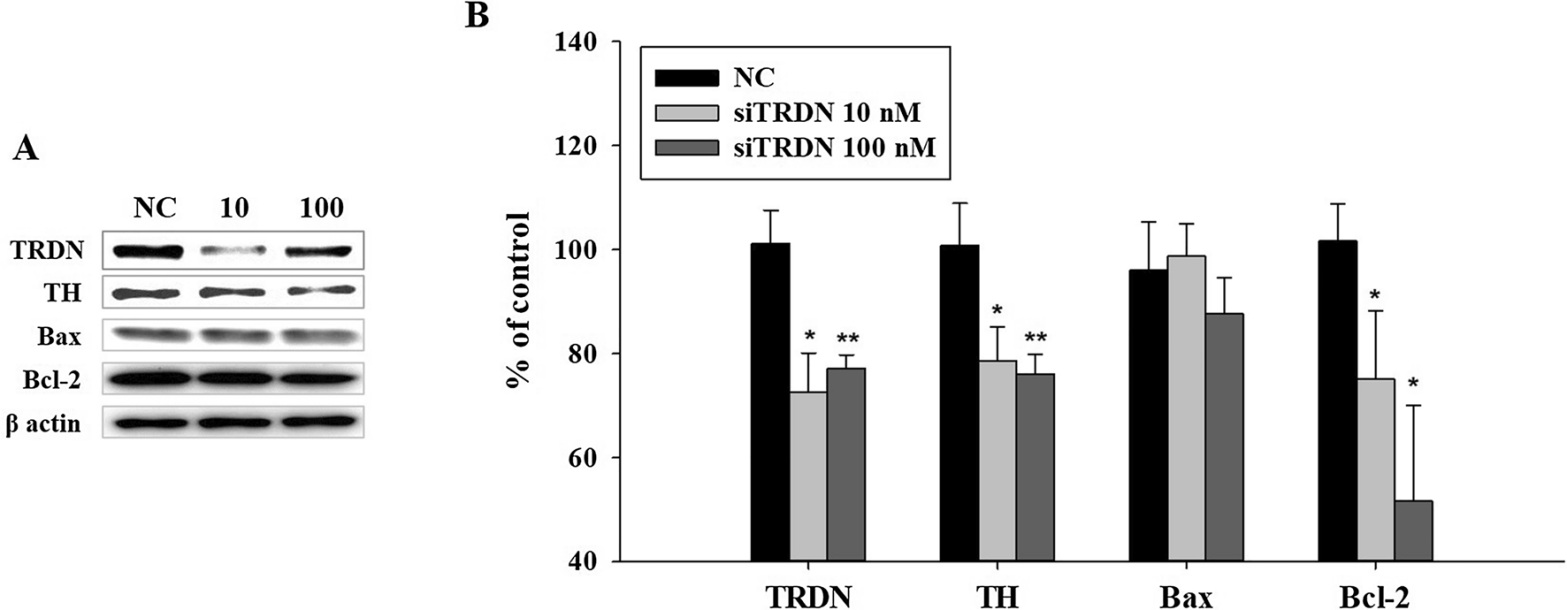
The SH-SY5Y cell line was transfected with TRDN siRNA(siTRDN) for 24 h. Negative control (NC) was transfected with 100 nM NC siRNA, and the others were transfected with 10- and 100-nM TRDN siRNA. The tyrosine hydroxylase (TH) level was consistently decreased in the 10- and 100-nM siTRDN treatment groups. The Bax level did not show significant changes, but the Bcl-2 level tended to decrease. The Bax/Bcl-2 ratio increased according to the decrease in TRDN level (*P < 0.05, **P < 0.005)

To observe the effect of TRDN alterations in human cells, SH-SY5Y cells were cultured and TRDN siRNA (siTRDN) was treated at concentrations of 10 and 100 nM for 24 h. After 24 h, the TH levels decreased in Fig. 4. This result showed that decreased TRDN induced a decrease in TH level. In addition to the expression levels of the proteins related with PD, to determine how TRDN reduction affects cells, the levels of Bcl-2-associated X (Bax) and Bcl-2 were assessed using WB. The results indicated that the Bax level did not change much but the Bcl-2 level decreased, and the Bax/Bcl-2 ratio increased in Fig. 4. From these results, we can expect that TRDN reduction causes apoptosis. This is important because it means that TRDN level reduction causes apoptosis in dopaminergic cells of PD (Additional file 1).

### Evaluation of cell death according to TRDN siRNA concentration

**Fig. 5:**
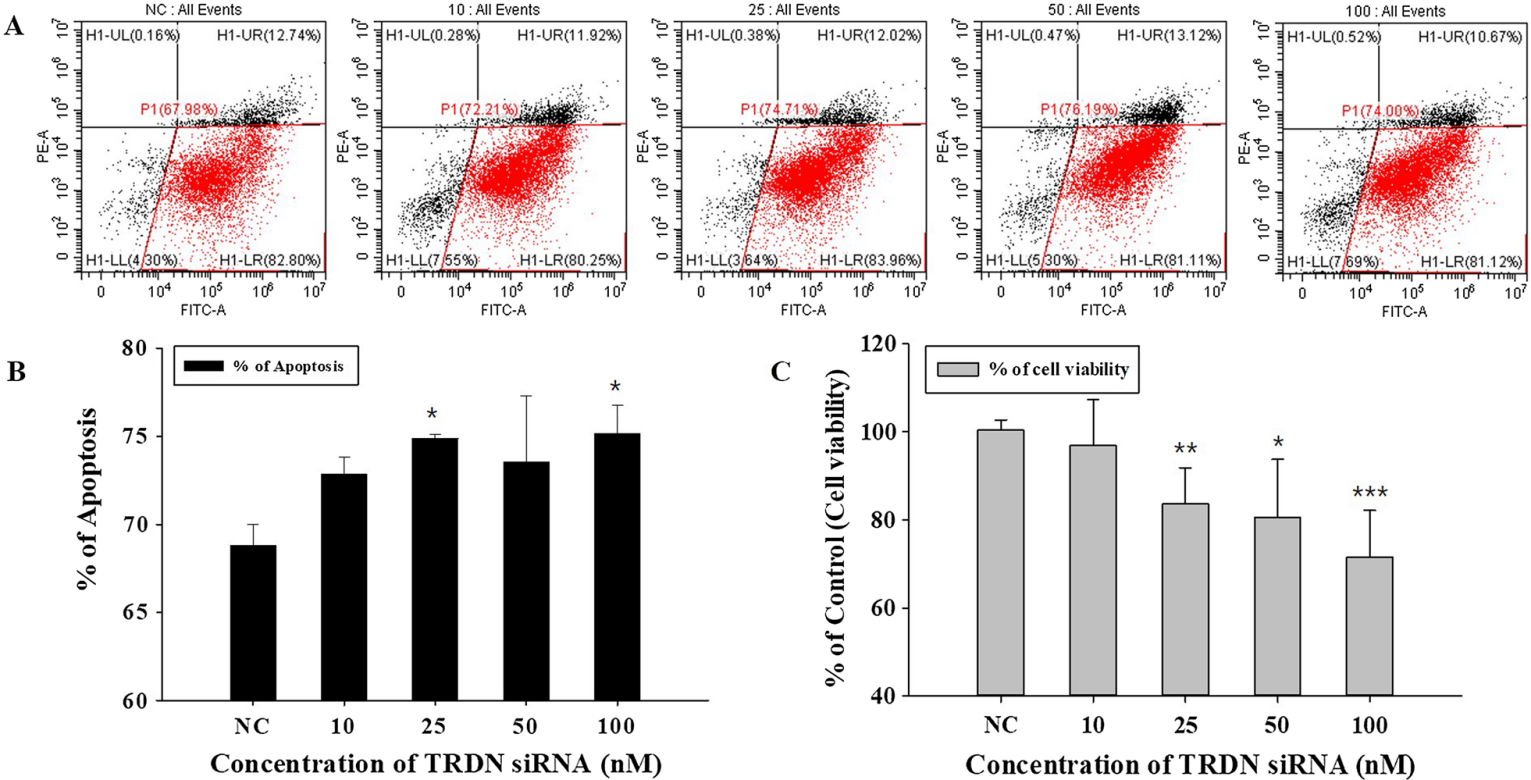
Fluorescence-activated cell sorting (FACS) analysis of the apoptosis of dopaminergic SH-SY5Y cells and cytotoxicity assay analysis according to various siTRDN concentrations.** A** Annexin V-FITC (horizontal axis) and propidium iodide (vertical axis) staining results are shown in the dot plots. The FACS dot plots in the negative control (NC 100 nM siRNA) and 10-, 25-, 50-, and 100-nM siTRDN treatment groups. The H1-LR zone is analyzed as an apoptosis zone. **B** Percent of plots in the H1-LR zone are shown in a bar graph, indicating apoptosis. As the treatment concentration of siTRDN increases, apoptosis also increases (*P < 0.05). **C** Cytotoxicity assay analysis to show the same cell viability as in the **B** graph groups. Cell viability decreased from 25 nM of siTRDN significantly (*P < 0.05, **P < 0.005, ***P < 0.0005)

To check for cell death, cytotoxicity assay was performed, and the result showed the cell viability depicted in Fig. 5C. We found no significant change in cell viability at 10-nM siTRDN, but the cell viability decreased from siTRDN concentrations > 25 nM (Fig. 5C). In 100-nM siTRDN, cell viability was reduced by 71.50%.

To more accurately confirm that cell death is due to apoptosis, cells were analyzed using a flow cytometry assay. Phosphatidylserines (PSs), which is known as an apoptosis signal factor in cells, were stained with Annexin V-Fluorescein (FITC), and a high FITC signal means that the cells are undergoing apoptosis. On the other hand, nuclear was stained with propidium iodide (PI), and lower PI signal means that the cells are still alive. Therefore, the H1-LR zone, high FITC signal, and low PI signal were confirmed to be useful for detecting apoptosis in the negative controls (100-nM NC siRNA treatment), and the 10-, 25-, 50-, 100-nM siTRDN treatments were effective for the SH-SY5Y cells (Fig. 5A). The percentages of the results are represented by the bar graph in Fig. 5B. The increase in apoptosis was significant from 25-nM siTRDN and consistently increased until 100-nM siTRDN. These results are semantically consistent with the cytotoxicity assay results that showed cell viability significantly affects apoptosis from 25 nM siTRDN.

## Discussion

To date, the function and role of TRDN in neurons are unknown. However, in this study, the expression pattern of TRDN in the SN region of the brain was observed and confirmed. It is interesting that the location of the expression of TRDN coincides with the SN region with dopaminergic cells. This suggests the possibility of a close interaction between TRDN and PD since the expression of TRDN decreased in the MPTP-induced PD mouse model. It was previously reported that TRDN expression in skeletal muscles decreases in a PD model [8]. In addition to the association between TRDN and PD in skeletal muscles, the similarity between the characteristics of TRDN and TH shown in the SN of the brain indicates a close association between TRDN and PD.

The TRDN level decreased in the MPTP-induced Parkinson’s disease model mouse, and the SH-SY5Y cells showed the characteristics of PD when *TRDN* was knocked down. In other words, decreased TRDN level induced decreased TH level as characteristics of PD. Through these, the relationship between TRDN level and PD was confirmed.

Furthermore, meaningful changes in the factors related to apoptosis in cells with reduced TRDN level were also associated with apoptosis. Our results showed that Bcl-2 decreased when the TRDN level decreased. Bcl-2, one of the anti-apoptotic proteins, readily inhibits cell death and apoptosis is regulated by Ca^2+^. Bcl-2 is known to regulate a release of Ca^2+^ from ER and contributes to mediating life or death of cells in between ER and mitochondria [14]. This means that we could deduce that TRDN is deeply related to the dopaminergic cell death in PD. In general, TRDN is already known to be a factor related to the inhibition of Ca^2+^ release from the SR in cardiac and general muscles [15–17]. Decreased TRDN level could be considered to induce the dysfunction of Ca^2+^ regulation in the ER of neurons in the SN, and the apoptosis process associated with Ca^2+^ is activated. However, how Ca^2+^ regulation changes in neurons and the mechanism underlying the induction of apoptosis by TRDN are not still unknown. More research is needed in this regard.

In current study, TRDN expression is associated with PD in that the TRDN level was reduced in the MPTP-induced PD mouse model and the pathological features of PD were induced by the decreased TRDN level. In addition, cells began to die at concentrations > 25 nM of siTRDN. The cell death by the decreased TRDN expression level was apoptotic cell death because the changes in the levels of the apoptosis-related factors such as Bcl-2, Bax/Bcl-2 ratio, and PS show that apoptosis occurred. Furthermore, as the concentration of siTRDN increased, the level of apoptosis also increased. Therefore, TRDN is associated with apoptosis of neurons and related with apoptotic cell death in PD. Thus, in dopaminergic cell death in PD, TRDN administration may be a potential candidate for PD treatment, although more research is needed to confirm this.

## Conclusions

In this study, we confirmed that the expression pattern of TRDN almost coincides with the region of TH expressed in dopaminergic neurons of SN and the pathological features of PD were induced by the decreased TRDN level. This means that we could deduce that TRDN is deeply related to the dopaminergic cell death in PD. In addition, this study may contribute to advancing research on TRDN as a factor related to PD.

## Supplementary Information

**Additional file 1.** Western blots of the results.

## Data Availability

All data analyzed during this study are included in this published article and its Additional files.
