# Supplementary material for: Association of decreased triadin expression level with apoptosis of dopaminergic cells in Parkinson’s disease mouse model
Source: BMC Neurosci. 2021 Nov 4;22:65. doi: 10.1186/s12868-021-00668-7 (PMC8567705; doi:10.1186/s12868-021-00668-7)
Supplement: Supplementary file 1 — Additional file 1. Western blots of the results. [file 12868_2021_668_MOESM1_ESM.pptx]

## Slide 1
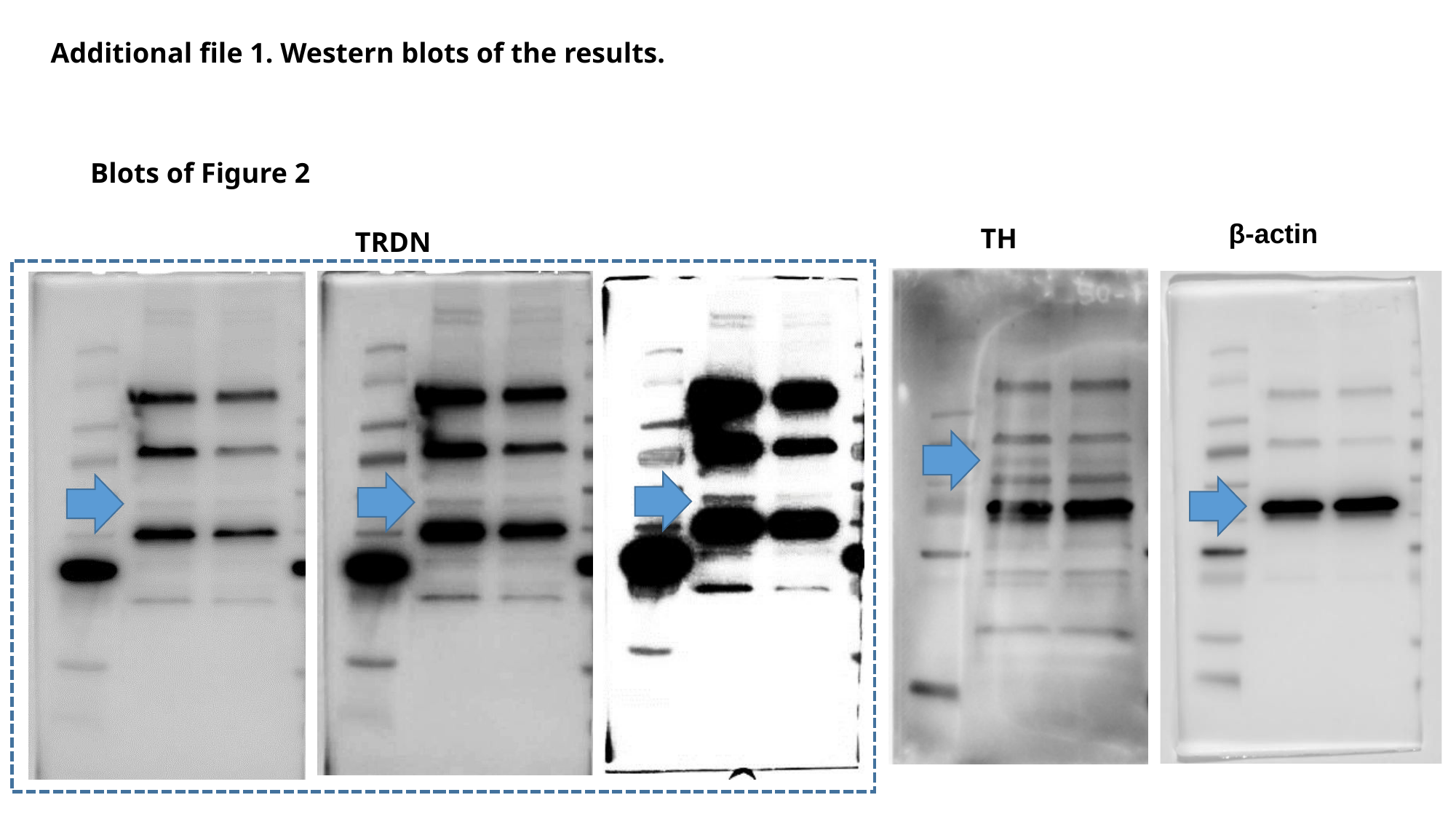

Additional file 1. Western blots of the results.
Blots of Figure 2
β-actin
TH
TRDN

## Slide 2
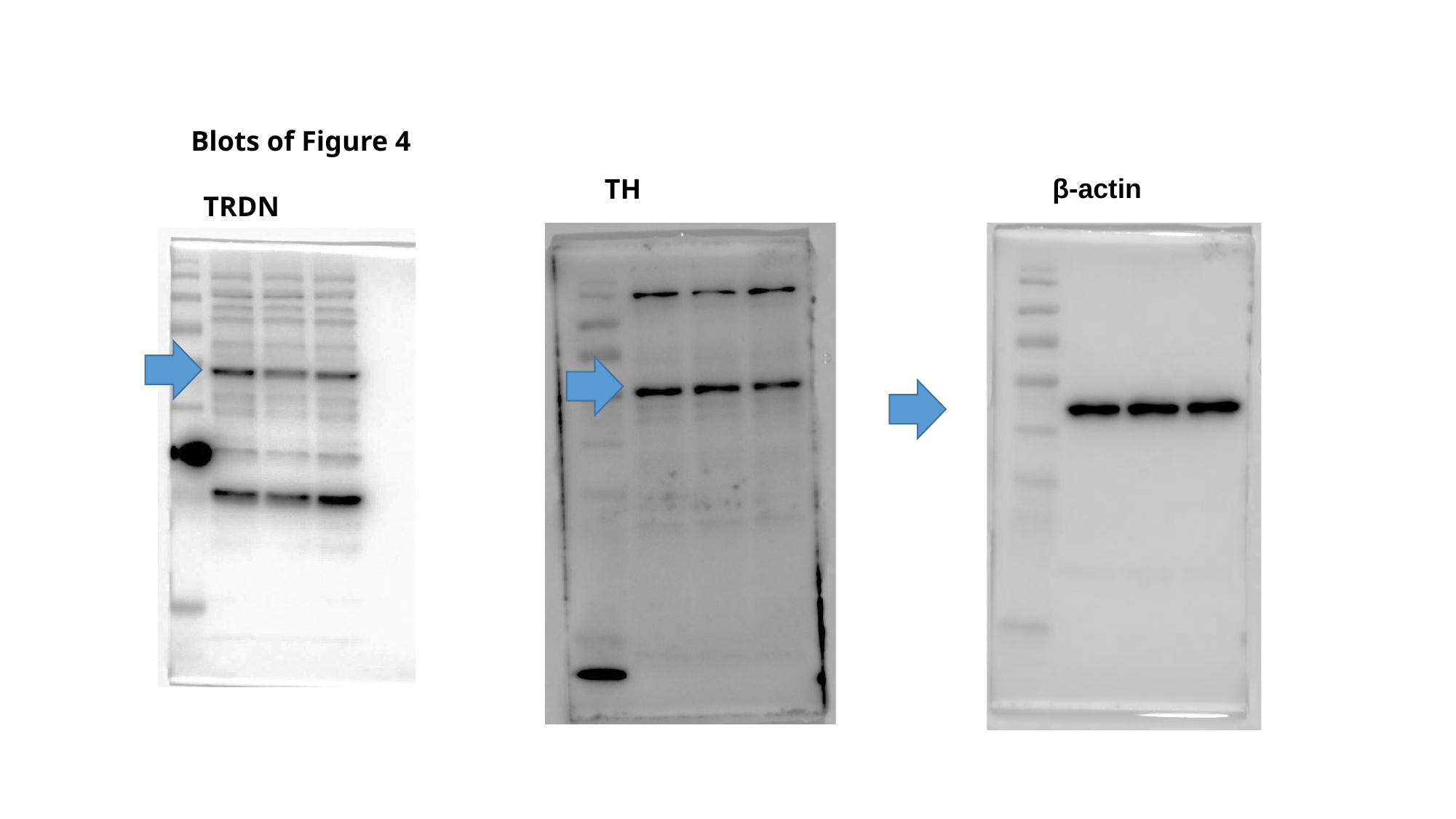

Blots of Figure 4
β-actin
TH
TRDN

## Slide 3
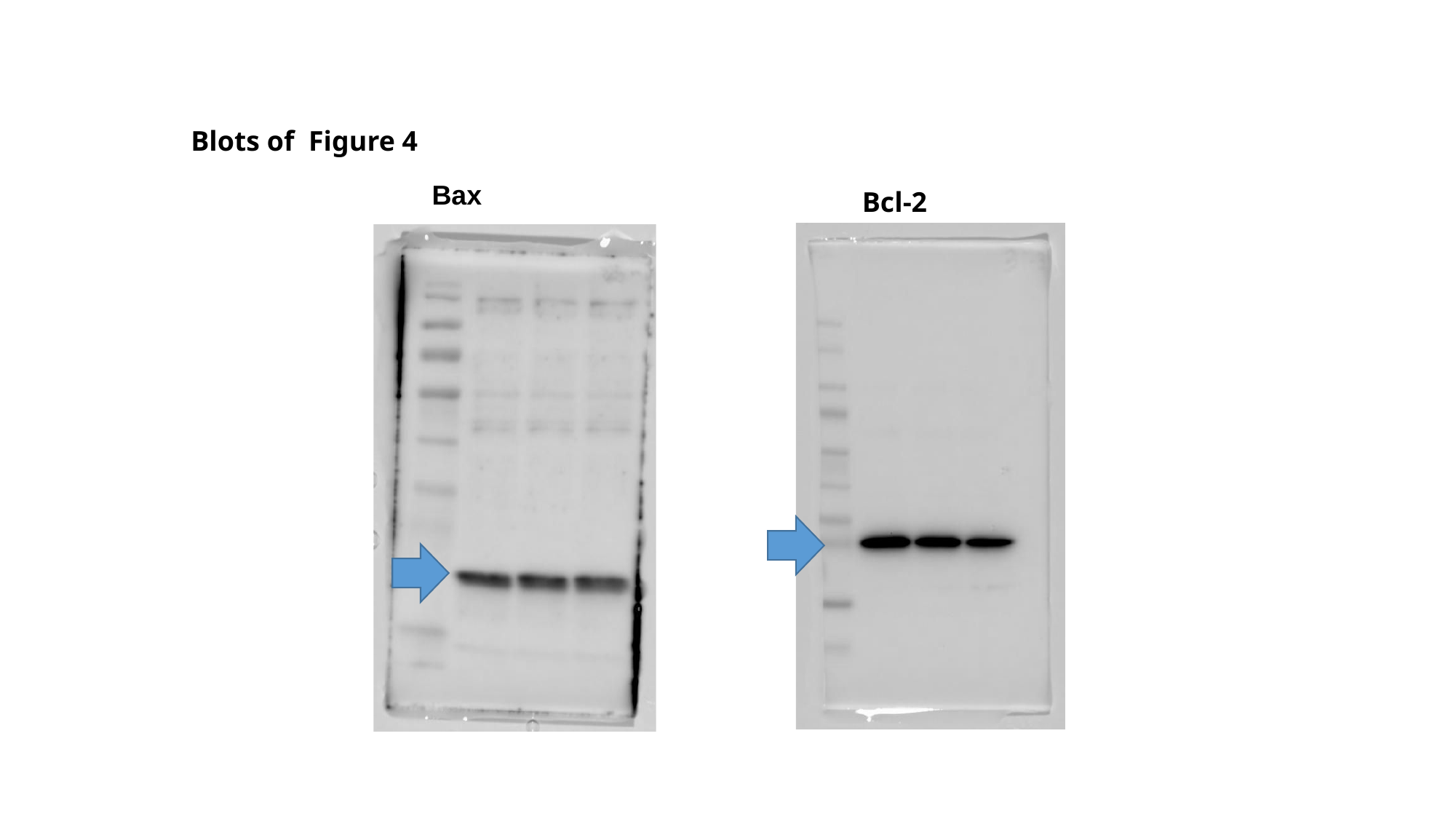

Blots of Figure 4
Bax
Bcl-2
